# Biofunctionalized Zinc Oxide Field Effect Transistors for Selective Sensing of Riboflavin with Current Modulation

**DOI:** 10.3390/s110706645

**Published:** 2011-06-27

**Authors:** Joshua A. Hagen, Sang N. Kim, Burhan Bayraktaroglu, Kevin Leedy, Jorge L. Chávez, Nancy Kelley-Loughnane, Rajesh R. Naik, Morley O. Stone

**Affiliations:** 1 711th Human Performance Wing, Human Effectiveness Directorate, Air Force Research Labs, Wright Patterson Air Force Base, OH 45433, USA; E-Mails: Jorge.ChavezBenavides.ctr.per@wpafb.af.mil (J.L.C.); Nancy.Kelley-Loughnane@wpafb.af.mil (N.K.-L.); Morley.Stone@wpafb.af.mil (M.O.S.); 2 Materials and Manufacturing Directorate, Air Force Research Labs, Wright Patterson Air Force Base, OH 45433, USA; E-Mails: SangNyon.Kim.ctr@wpafb.af.mil (S.N.K.); Rajesh.Naik@wpafb.af.mil (R.R.N.); 3 Sensors Directorate, Air Force Research Labs, Wright Patterson Air Force Base, OH 45433, USA; E-Mails: Burhan.Bayraktaroglu@wpafb.af.mil (B.B.); Kevin.Leedy@wpafb.af.mil (K.L.)

**Keywords:** aptamer, biomolecular detection, ZnO-FET, sensor, riboflavin, selectivity, label-free, biofunctionalization

## Abstract

Zinc oxide field effect transistors (ZnO-FET), covalently functionalized with single stranded DNA aptamers, provide a highly selective platform for label-free small molecule sensing. The nanostructured surface morphology of ZnO provides high sensitivity and room temperature deposition allows for a wide array of substrate types. Herein we demonstrate the selective detection of riboflavin down to the pM level in aqueous solution using the negative electrical current response of the ZnO-FET by covalently attaching a riboflavin binding aptamer to the surface. The response of the biofunctionalized ZnO-FET was tuned by attaching a redox tag (ferrocene) to the 3′ terminus of the aptamer, resulting in positive current modulation upon exposure to riboflavin down to pM levels.

## 1. Introduction

Development of sensing systems, whether they are for vapor or liquid states, small or large molecules, all need a scheme where highly selective detection can occur. Additionally, a reporting system must be designed to identify when the target molecule has been detected. One way of transducing target detection as an electrical signal is through the use of a field effect transistor (FET). Many FET structures utilize semiconductors that are highly sensitive to environmental changes, such as the presence of different chemical species in both liquid and vapor states [1–3], although with little to no selectivity. Different methods have been used to add selectivity to FET devices, such as controlling the gate voltage on metal-oxide nanowires [4], the addition of different materials such as polymers [5–8], small molecules [9], and membranes [10]. Another approach to impart selectivity is by taking inspiration from nature, by utilizing molecules such as antibodies, peptides, and oligonucleotide based (DNA or RNA) aptamers which are naturally designed to bind molecules with high specificity. These biorecognition elements have shown selective and sensitive detection in FETs with semiconducting nanostructures such as single walled carbon nanotubes [11–15] and silicon nanowires [16–19]. DNA aptamers are particularly powerful detection molecules, and through processes like the systematic evolution of ligands by exponential enrichment (SELEX) [20], aptamers can be selected to bind a wide variety of targets such as small molecules [21], proteins [22], DNA [23], and bacteria [14]. Additional flexibility in aptamer detection systems can be added by attaching redox molecules, such as ferrocene or methylene blue, to the 3′ end of the DNA oligomer. The secondary structure of the aptamer and the conformational change the aptamer undergoes upon target binding controls its distance from the surface from which is anchored. Thus, the distance of a redox molecule which is placed the end of the aptamer will be controlled by the length and conformational state of the aptamer. This flexible aptamer detection technique has been well studied by Plaxco *et al*. [24–26] in electrochemical analysis. Biofunctionalized transistor-based sensing has also been studied with various detection mechanisms including; (1) surface conductance of semiconducting channel [16], (2) electrostatic gating, in which gate potential is disturbed by ions near the semiconductor [27], (3) carrier mobility change [28,29], (4) interfacial contact effect [28,30], (5) permittivity change [28,31] and Schottky gate modulation [32]. With those various detection mechanisms and strategies, the typical simplified transistor-based sensor operates in a mode where the binding event between the device anchored receptor and target is reported as a change in conductance. In this case, we demonstrate the primary sensing response of the small molecule riboflavin using the riboflavin-binding aptamer on a ZnO-FET platform which can be controllably acquired by using the native negative charge in the DNA backbone and its redox chemical pendant molecule to modulate the current response.

## 2. Experimental Section

### 2.1. Materials

Riboflavin aptamers were synthesized and purchased from Integrated DNA Technologies Inc. with HPLC purification and the following sequence: 5′-SH-AGA GAG GAA CGA CGG TGG TGG AGG AGA TCG TTC C-3′. Ferrocene terminated riboflavin aptamers were purchased from Friz BioChem (Germany) and HPLC purification with the following sequence: 5′-SH-AGA GAG GAA CGA CGG TGG TGG AGG AGA TCG TTC C-Ferrocene-3′. Riboflavin (CAS 83-88-5) and 2-quinoxaline carboxylic acid (CAS 879-65-2) were purchased from Sigma-Aldrich and used without further purification. Sterile water (UltraPure distilled DNAse RNAse free) was purchased from Invitrogen. The silane coupling agent, 3-glycidoxypropyl dimethylethoxysilane, was purchased from Gelest Inc. and used without further purification. The ZnO FET devices were a bottom-gate design on a silicon wafer with a 30 nm silicon dioxide (SiO_2_) layer as the gate insulator and deposited via plasma enhanced chemical vapor deposition (PECVD). ZnO was deposited on top of the SiO_2_ using a Neocera Pioneer 180 pulsed laser deposition system using a KrF eximer laser with a thickness of 100 nm. Source/drain contacts of titanium/platinum/gold (20/30/350 nm) were deposited by evaporation and liftoff techniques.

### 2.2. Methods

A 3” wafer of ZnO FETs was diced into 15 mm × 5 mm individual FET arrays (>10 transistors) for functionalization and sensor testing. The diced FET arrays were first cleaned with an ethanol and deionized water wash followed by drying in nitrogen. The silane solution was prepared by adding 650 μL of 3-glycidoxypropyl dimethylethoxysilane to 50 mL of a 95% EtOH 5% water solution (UltraPure water). This solution was mixed on a stir plate at room temperature for 5 min for the silane to hydrolyze and polymerize. The diced FET arrays were then placed in the silane solution with gentle shaking for 2 min and promptly washed with ethanol and dried with nitrogen. The curing step took place in a vacuum oven for 10 min at 100 °C. The aptamer solutions were made at a concentration of 12 μg/mL in UltraPure water in a 1.5 mL sterile plastic vial. The diced FETs were placed in the vial and gently shaken for 5 min and promptly washed with UltraPure water and dried with nitrogen. The ZnO FETs at this point were now fully functionalized with a riboflavin aptamer (either ON state or OFF state) and ready for sensor testing (preferably same day as functionalization).

The ZnO AptaFETs were tested individually for response to the target (riboflavin) or negative control (2-quinoxaline carboxylic acid) using a Keithley SCS 4200 Semiconductor Analyzer and probe station. A 20 μL drop of UltraPure water was placed on the FET being tested and the current *vs*. voltage (I–V) response monitored until it stabilized (∼2 min). After this point, an additional 20 μL drop of the target or negative control solution was placed on the FET. The response was monitored as a gate voltage sweep, where the source drain voltage (V_SD_) is constant at 0.2 V and gate voltage (V_G_) is modulated from −5 V ∼ +15 V immediately after exposure to the solution. When monitored as a real-time response, a constant V_SD_ and V_G_ are applied to the FET and the source/drain current was recorded at one data point per second while various solutions are applied to the FET. Again, a 20 μL drop of UltraPure water was placed on the FET before target or negative control exposure.

## 3. Results and Discussion

### 3.1. Mechanism of Transduction

**Figure 1.**
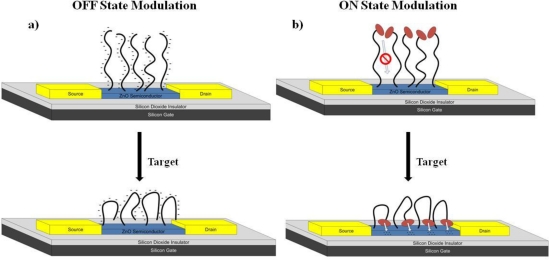
Schematic of aptamer binding conformation and transduction mechanism for: (**a**) OFF state AptaFET, (**b**) ON state current modulation along with corresponding aptamer conformation change upon target binding.

When an aptamer binds its target, it adopts a secondary conformation, which in many cases involves a looped and more compact structure, as is the case with the riboflavin aptamer [33] (see supplemental information for the secondary structure of the riboflavin aptamer as obtained by using the mfold software [34]). This secondary structure brings the negative charges from the DNA backbone closer to the surface of the semiconductor, and has a negative top gating effect on the device (termed as an OFF state AptaFET). For an n-type semiconductor, like ZnO, this binding event will result in a decrease in current due to this negative gating effect, and is shown schematically in Figure 1(a). The negative electrical response (decreasing current) upon binding of riboflavin can be modified to exhibit a positive response (increasing current) by the addition of an electron donating molecule, such as ferrocene to the 5′ end of the DNA oligomer. In this scheme, the secondary structure of the aptamer upon binding of riboflavin causes the ferrocene tag to come into close contact with the ZnO semiconductor, shown schematically in Figure 1(b). This allows for the injection of electrons from the ferrocene molecules into the n-type ZnO semiconductor causing an increase in current upon riboflavin binding—termed the ON state AptaFET.

### 3.2. Biofunctionalization of ZnO FET

**Figure 2.**
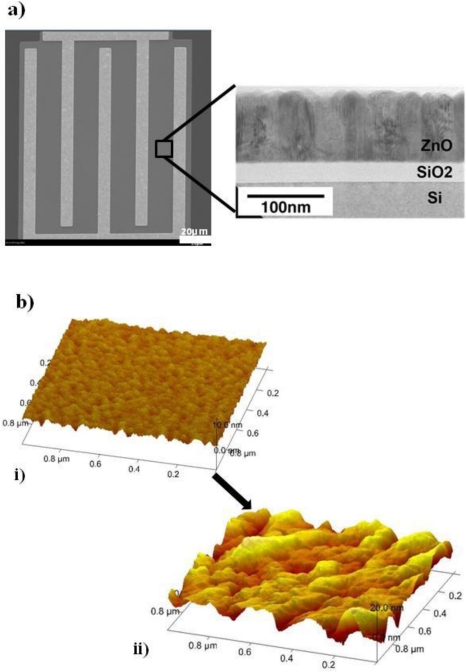
ZnO-FET electron and atomic force microscopy: (**a**) scanning electron micrograph of the interdigitated electrode FET configuration and transmission electron micrograph of the FET cross-section, and (**b**) atomic force micrograph after (i) silane linker functionalization and after (ii) aptamer functionalization via silane linker.

A promising semiconducting material in high frequency thin film transistors is ZnO [35]. Pulsed laser deposition (PLD) is used to deposit ZnO for FET fabrication, and is a room temperature technique which is compatible with a wide variety of substrates, including plastic flexible substrates [36]. ZnO grows into vertically aligned ZnO nanostructures through PLD deposition [Figure 2(a)]. The nanostructured surface morphology creates a large effective surface area, making it an ideal candidate for use in FET sensors. The ZnO FET devices are based on an interdigitated design with a 10 μm gap [Figure 2(a)].

Our ZnO Aptamer-FET sensor is designed to detect riboflavin, which is a physiological indicator also known as vitamin B2, and has a well studied aptamer sequence [33]. We use the riboflavin binding aptamer with the sequence 5′-SH-AGA GAG GAA CGA CGG TGG TGG AGG AGA TCG TTC C-3′ which includes a thiol group at the 5′ end for the chemical functionalization with ZnO surface. An additional 5 bp (AGAGA) spacer is integrated into the aptamer sequence to prevent the active binding pocket from steric hindrance due to nonspecific adsorption to the ZnO surface. A silane coupling agent, 3-glycidoxypropyl dimethylethoxysilane, was used to covalently link the aptamer to the ZnO semiconductor. The silane contains three alkoxy groups which are hydrolyzed in ethanol followed by condensation into oligomers which hydrogen bond with the hydroxyl groups on the ZnO surface. Drying and curing creates a covalent linkage of the silane to the surface (schematic shown in supplemental information Figure S1) [37]. The ZnO-FET is incubated in the silane solution (2.5% in 95% EtOH and water) for 2 min, washed in ethanol, dried in nitrogen, and annealed at 100 °C in a vacuum oven for 10 min. The ZnO-FETs are then incubated in the riboflavin DNA aptamer for 5 min at a concentration of 12 μg/mL in sterile water, rinsed with sterile water, and dried with nitrogen. AFM measurements on the ZnO-FET surface [Figure 2(b)] show significant surface morphology changes associated with the silane and aptamer attachment with surface roughness increasing from 0.59 nm (silane) to 4.01 nm (silane + aptamer).

Additionally, x-ray photoelectron spectroscopy experiments show the presence of nitrogen atoms on the DNA functionalized ZnO FETs, whereas no nitrogen is present on the unfunctionalized FETs (shown in supplemental information Figure S2). Nitrogen atoms are found in abundance in DNA in both the backbone and base structures.

### 3.3. Device and Sensor Characterization

**Figure 3.**
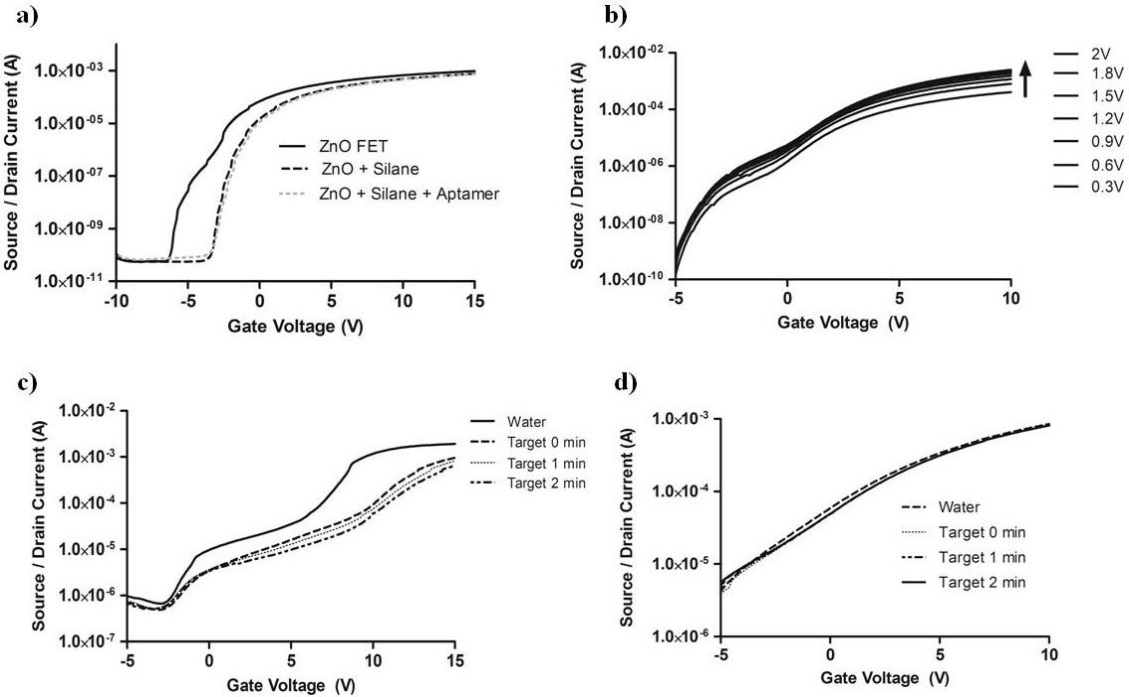
AptaFET device performance: (**a**) Source/Drain (I_SD_) current *versus* Gate Voltage (V_G_) at a Source/Drain Voltage (V_SD_) = 0.5 V for unfunctionalized ZnO-FET, ZnO-FET with silane linker, and ZnO-FET with silane linker and aptamer, (**b**) I_SD_ *vs*. V_G_ with modulation of V_SD_. (**c**) I_SD_ *vs*. V_G_ for conditions of water equilibrium, immediately after exposure of 10 μM riboflavin, and time points of 1 min and 2 min after exposure. (**d**) Response of an unfunctionalized ZnO-FET after exposure to 10 μM riboflavin.

The high ON/OFF ratio (>10^7^) of a ZnO-FET enables one to obtain excellent baseline device reproducibility. As seen in Figure 3(a), biofunctionalization with the aptamer does not interrupt the high ON/OFF ratio, where the gate voltage modulation data is presented for the same ZnO transistor in the native state, silane treated, and aptamer functionalized. The threshold voltage increases after each functionalization step due to the addition of electrically resistive materials to the surface of the semiconductor while maintaining the 10^7^ ON/OFF ratio. Modulation of the source/drain voltage along with the gate voltage is shown for the riboflavin AptaFET in Figure 3(b) with high device reproducibility (see supplemental Figure S4).

For these riboflavin AptaFET devices, a drop of sterile water is placed on the device to rehydrate the covalently bonded aptamers and the current *versus* voltage (I–V) curve is monitored until the current running through the semiconductor is stabilized. Once reaching a steady state current, the AptaFET is exposed to different concentrations of the target molecule for sensor analysis. Figure 3(c) shows that the source/drain current (V_SD_ = 0.2 V) decreases upon the sensing of 10 μM aqueous riboflavin throughout the modulated gate voltage of −5 ∼ +15 V while the bare silanated ZnO-FET shows negligible response to the same target [Figure 3(d)]. The immediate (0 min) decrease in current after exposure to the riboflavin target appears to only slightly change at subsequent gate voltage sweeps (1 and 2 min), showing that a majority of the binding events happen instantly upon addition of the target.

**Figure 4.**
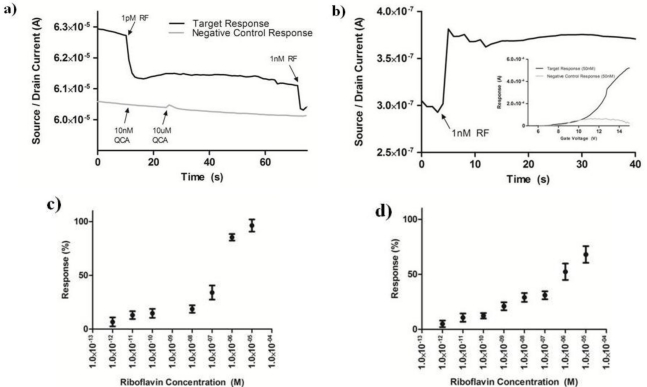
Sensor performance: (**a**) Real-time measurement of OFF state sensor response to the target riboflavin and negative control QCA via I_SD_ *vs*. Time (s) with V_SD_ = 0.2 V and V_GS_ = 8 V. (**b**) Real-time ON state sensing via I_SD_ *vs*. time (s) for 10 nM exposure to riboflavin. Inset shows I_SD_ *vs*. V_G_ sensor response to 10 μM riboflavin and 10 μM negative control QCA. (**c**) Signal dependence *vs*. riboflavin concentration (M) for OFF state sensing scheme. (**d**) Signal dependence *vs.* riboflavin concentration (M) for ON state sensing scheme.

In a real-time mode, where the source/drain voltage (V_SD_) and gate voltage (V_G_) are set at a constant value, and the source/drain current (I_SD_) is monitored *versus* time, the riboflavin AptaFET shows a maximal response at V_G_ of 6–10 V at V_SD_ of 0.2 V (see supplemental Figure S5). The exact maximum response value varies from device to device, most likely due to the variability in aptamer density on the FET, but is in the range of 6–10 V. For the real time riboflavin sensing, we set V_G_ = 8 V and V_SD_ = 0.2 V. After reaching equilibrium with a 20 μL drop of sterile water, an additional 20 μL drop containing the target solution is placed on the AptaFET and the current change is monitored in real time. An instant current drop of 140 nA is obtained upon exposure to 1 pM riboflavin [Figure 4(a)]. Further addition of 1 nM riboflavin after 60 s shows a response from the AptaFET as observed by an additional decrease in current. Control experiments, where the AptaFET was exposed to various concentrations (10 nM and 10 μM) of a chemical analogue to riboflavin, 2-quinoxaline carboxylic acid (QCA), shows negligible response [Figure 4(a)].

Figure 4(c) shows riboflavin concentration as a function of AptaFET current response calculated as the percentage change in current with respect to the equilibrated baseline. The lowest overall current response is on the order 100 nA, well above the noise level of the semiconducting analyzer. The device response for each concentration was assessed using the real time detection mode described above, and calculated as the percentage change between the two plateaus seen in Figure 4(a). The overall sensitivity of this OFF state AptaFET is 1 pM of riboflavin in water. Although the 140 nA response was above the noise level for detection, concentrations lower than 1 pM did not show any current response. Figure 4(b) shows the real-time response of the ON state AptaFET structure, which shows immediate increase in current with the addition of 1 nM riboflavin in water. The inset in Figure 4(b) shows the preferential response of riboflavin compared to the negative control small molecule QCA. The current response *versus* riboflavin concentration shows good correlation and sensing down to 1 pM, which is comparable to the detection limit of the OFF state AptaFET scheme.

The advantage to the ON state detection scheme is seen in the more controllable signal response as a function of riboflavin concentration [Figure 4(d)] as compared to the OFF state devices [Figure 4(c)]. Additionally, the ability to modulate the current between an ON and OFF state adds flexibility to this sensor system. Each aptamer has a unique sequence, and forms a unique secondary structure upon binding of its target. In some structures, it will be advantageous to analyze both the ON and OFF state to determine any sensitivity increases. Running both the ON and OFF state BioFETs in parallel for the same target would also provide a method for assessing false positive and negative responses. This is important for applications such as medical diagnostics, which require reliable concentration based detection systems [38].

## 4. Conclusions

In conclusion, single stranded DNA aptamers provide a selective binding motif for sensing in a liquid state for small molecules like riboflavin. The secondary structure adopted by the aptamer upon binding of its target, in this case riboflavin, can be transduced as an electrical signal due to the rearrangement of negative charges on the backbone of the DNA oligomer into a more compact structure on a semiconductor surface. This transduction is possible by immobilizing the aptamer covalently on the surface of a zinc oxide field effect transistor. The electrical current response can be modulated to provide either an increase or decrease in current upon binding by the presence or absence (respectively) of a ferrocene redox tag on the 3′ end of the aptamer. Both schemes of current response resulted in selective sensing of riboflavin in water to pM levels, with greater control over signal response in the ON state system.

The novelty in this aptamer based sensor system is the semiconductor ZnO which has two significant advantages. First, ZnO is deposited via pulsed laser deposition which operates at room temperature with no further thermal annealing necessary. This enables the use of this material on flexible (plastic) substrates, which is not possible with typical semiconductor materials in aptamer based sensors such as silicon nanowires and carbon nanotubes. Second, ZnO naturally forms a unique nanostructure upon pulsed laser deposition which provides a large surface area for molecular detection without the need for further processing which is required for patterned nanowires. The direct detection signal strategy acquired in this study resulted in selective detection down to low concentration (pM) levels which is comparable to state of the art aptamer based sensors, with the added benefit of a ZnO-FET platform with significant fabrication advantages.

## Supplementary Materials
