# Supplementary material for: Biofunctionalized Zinc Oxide Field Effect Transistors for Selective Sensing of Riboflavin with Current Modulation
Source: Sensors (Basel). 2011 Jun 27;11(7):6645–55. doi: 10.3390/s110706645 (PMC3231651; doi:10.3390/s110706645)

Supplementary Information

Figure S1. Schematic of silane linker and aptamer attachment on a ZnO surface. (**a**) Surface condition and silane oligomer structure, (**b**) covalent attachment of silane oligomer to ZnO surface, (**c**) covalent attachment of aptamer to silane linker and complete functionalized structure.

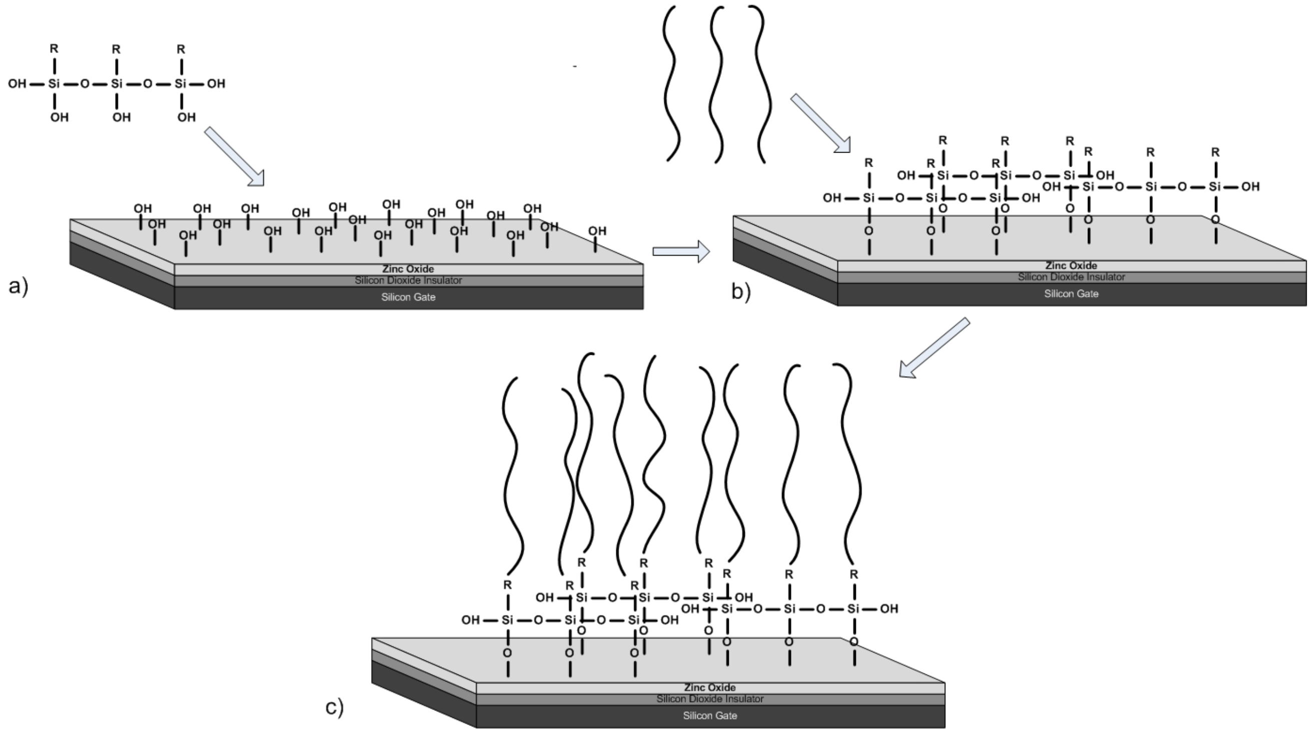

Figure S2. X-ray photoelectron spectroscopy data confirming attachment of silane linkage and aptamers to the ZnO semiconductor. ZnO FET shows on Nitrogen, ZnO FET w/Silane shows an increase in Si 2s and 2p, and AptaFET shows the presence of 0.64% Nitrogen associated with the large amount of Nitrogen present in DNA.

**Sample**	Si 2s	Si 2p	N 1s	C 1s	O 1s
**ZnO FET**	17.15%	16.42%	-	7.55%	30.83%
**ZnO FET w/Silane**	17.44%	17.34%	-	7.81%	31.44%
**AptaFET**	17.48%	17.10%	0.64%	6.98%	31.33%

Figure S3. Secondary structure of riboflavin aptamer as obtained by mfold[33].

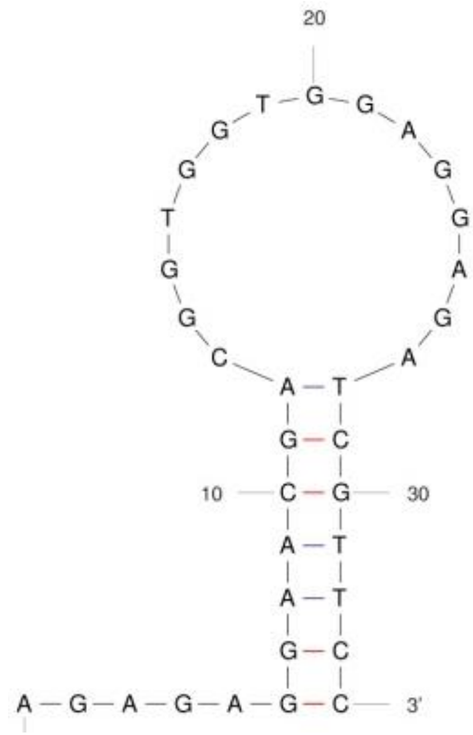

Figure S4. Device reproducibility via I_{SD} *vs*. V_G for three aptamer functionalized ZnO-FETs.

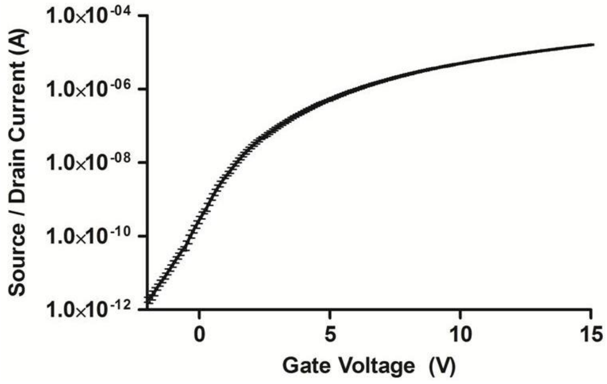

Figure S5. Gate voltage modulation response of ON state AptaFET to 10 µM riboflavin immediately after exposure (0 min) and after 1 min. Response increases between 6–10 V with a maximum at 8 V.

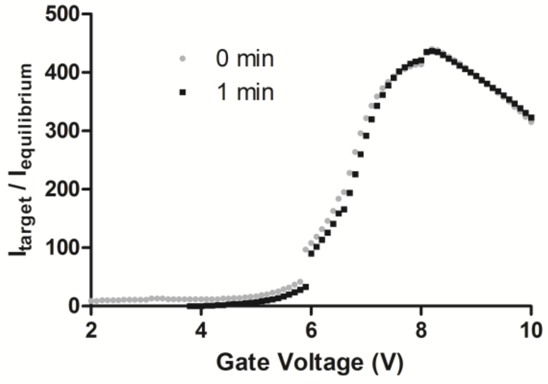

Supplement: Supplementary file 1 [file sensors-11-06645-s001.pdf]
